# Publisher Correction: Cryo-EM structures of type IV pili complexed with nanobodies reveal immune escape mechanisms

**DOI:** 10.1038/s41467-024-47081-2

**Published:** 2024-04-03

**Authors:** David Fernandez-Martinez, Youxin Kong, Sylvie Goussard, Agustin Zavala, Pauline Gastineau, Martial Rey, Gabriel Ayme, Julia Chamot-Rooke, Pierre Lafaye, Matthijn Vos, Ariel Mechaly, Guillaume Duménil

**Affiliations:** 1 Institut Pasteur, Université Paris Cité, INSERM UMR1225, Pathogenesis of Vascular Infections, 75015 Paris, France; 2 Institut Pasteur, Université Paris-Cité, CNRS, UAR 2024, Mass Spectrometry for Biology, 75015 Paris, France; 3 Institut Pasteur, Université Paris-Cité, CNRS-UMR 3528, Antibody Engineering Platform, 75015 Paris, France; 4 NanoImaging Core Facility, Center for Technological Resources and Research, Institut Pasteur, 75015 Paris, France; 5 Institut Pasteur, Crystallography Platform-C2RT, CNRS-UMR 3528, Université Paris Cité, Paris, France; 6 Present Address: Sanofi R&D, Integrated Drug Discovery, CRVA, 94403 Vitry-sur-Seine, France

**Keywords:** Cryoelectron microscopy, Bacterial pathogenesis, Bacterial immune evasion, Post-translational modifications

Correction to: *Nature Communications* 10.1038/s41467-024-46677-y, published online 18 March 2024

The original version of this Article contained an error in the Abstract, which incorrectly read ‘Cyro-EM in combination with molecular dynamics simulation of the nanobody-pilus complexes reveals how the different types of pili surface modifications alter nanobody binding.’ The correct version states ‘Cryo-’ in place of ‘Cyro-’.

The original version of this Article contained an error in the Introduction, which incorrectly read ‘Finally, a third level of variation comes from another PTM on serine 68, phosphoforms that can be phosphocholine (PC) or phosphoethanolamine (PEA) if the pptA allele is present in the strain, or a phosphoglycerol (glycerol-3-phosphate or G3P) if the pptB allele is present.’ The correct version states ‘serine 69’ in place of ‘serine 68’.

The original version of this Article contained an error in Figure 1 legend, which incorrectly read ‘c. Individual pilin subunit with G3P and GATDH, the hypervariable loop appears in dark blue. Close-ups of the phosphoglycerol moiety on S69 and on the GATDH moiety present on S63 are indicated.’ The correct version adds ‘SB’ after ‘Individual’.

The original version of this Article contained an error in the Results section, which incorrectly read ‘In contrast, despite the minor difference between the two sugar forms, the presence of a DATDH had a strong impact on F10 binding in both assays (Fig. 3b and Fig. 7c).’ The correct version states ‘3a’ in place of ‘3b’.

The original version of this Article contained three errors in the Methods section, which incorrectly read ‘ΔG4 strain (GATDH). The pglB1 ORF without the first 23 bp was amplified with Phusion Plus DNA Polymerase (Thermo Scientific, #F630S) from MS11 chromosomal DNA using the primers pglB1_MS11_fwd and pglB1_MS11_rev.’, ‘GC agar plates’ and ‘100x oil’. The correct version adds ‘(Supplementary Table 2)’ after ‘pglB1_MS11_rev.’, and states ‘GCB agar plates’ in place of ‘GC agar plates’ and ‘40x’ in place of ‘100x’.

These have been corrected in both the PDF and HTML versions of the Article.

