# Supplementary material for: Cryo-EM structures of type IV pili complexed with nanobodies reveal immune escape mechanisms
Source: Nat Commun. 2024 Mar 18;15:2414. doi: 10.1038/s41467-024-46677-y (PMC10948894; doi:10.1038/s41467-024-46677-y)

a

Structural variation of pili surface								
	**Pilin sequence**			**Glycosylation**			**Phosphorylation**	
**Genes involved**	Pilin silence cassettes (*pilS*)			Glycosylation enzymes (*pglB1/2*)		Second sugar (*pglA*)	Pilin phosphotransferase (*pptA/B*)	
**Mechanism of variation**	Antigenic variation			Presence of specific allele		Phase variation	Presence of specific allele	
**Surface structures**	Others	SA	SB	GATDH	DATDH	G/DATDH-Hexose	G3P	PEA
**F10 binding**	?							?
**C24 binding**	?							?

b

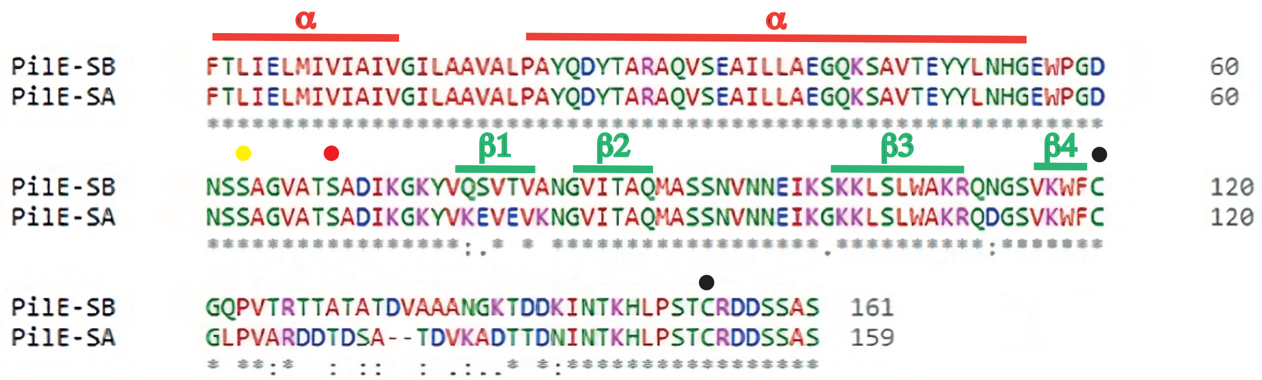

c

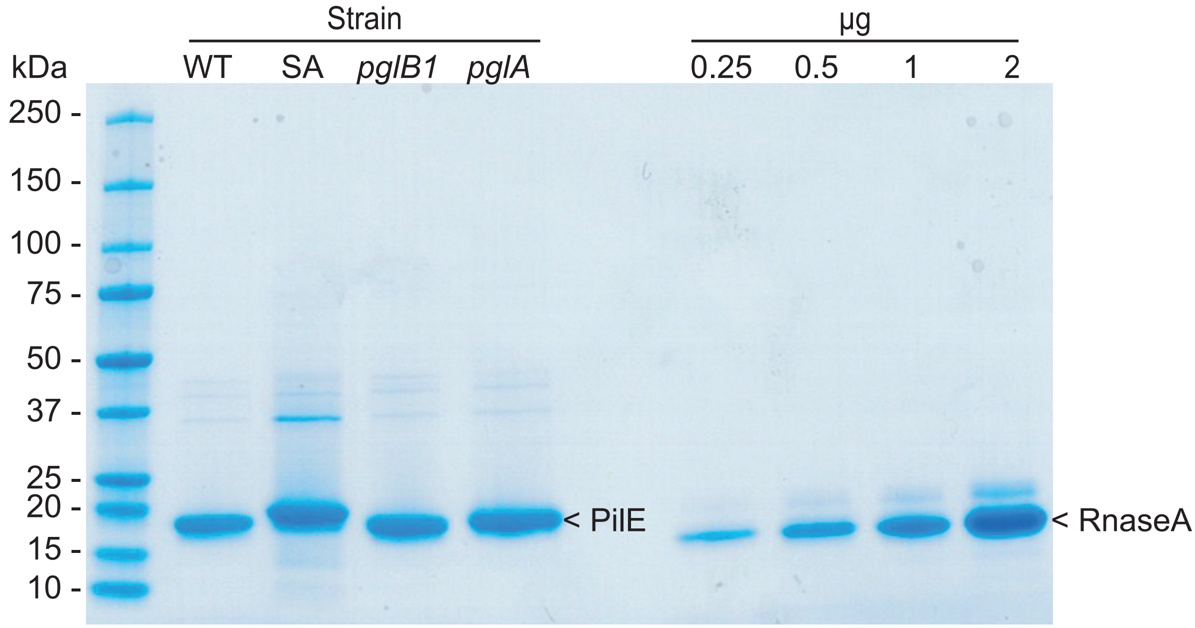

Supplementary Fig. 1: Pilus surface diversity and pilus purification. a, Sources of pilus surface variation, variation mechanisms, and their genetic basis. b, Sequence alignment of the PilE protein expressed by the SA and SB sequence types. The dots indicate the glycosylation site (yellow), the G3P modification (red) and cysteines involved in disulfide bond formation (black). c, SDS-PAGE and Coomassie staining of pilus preparations used in this study. Different amounts of purified RNAseA (Cytiva) were used as a reference for quantification.

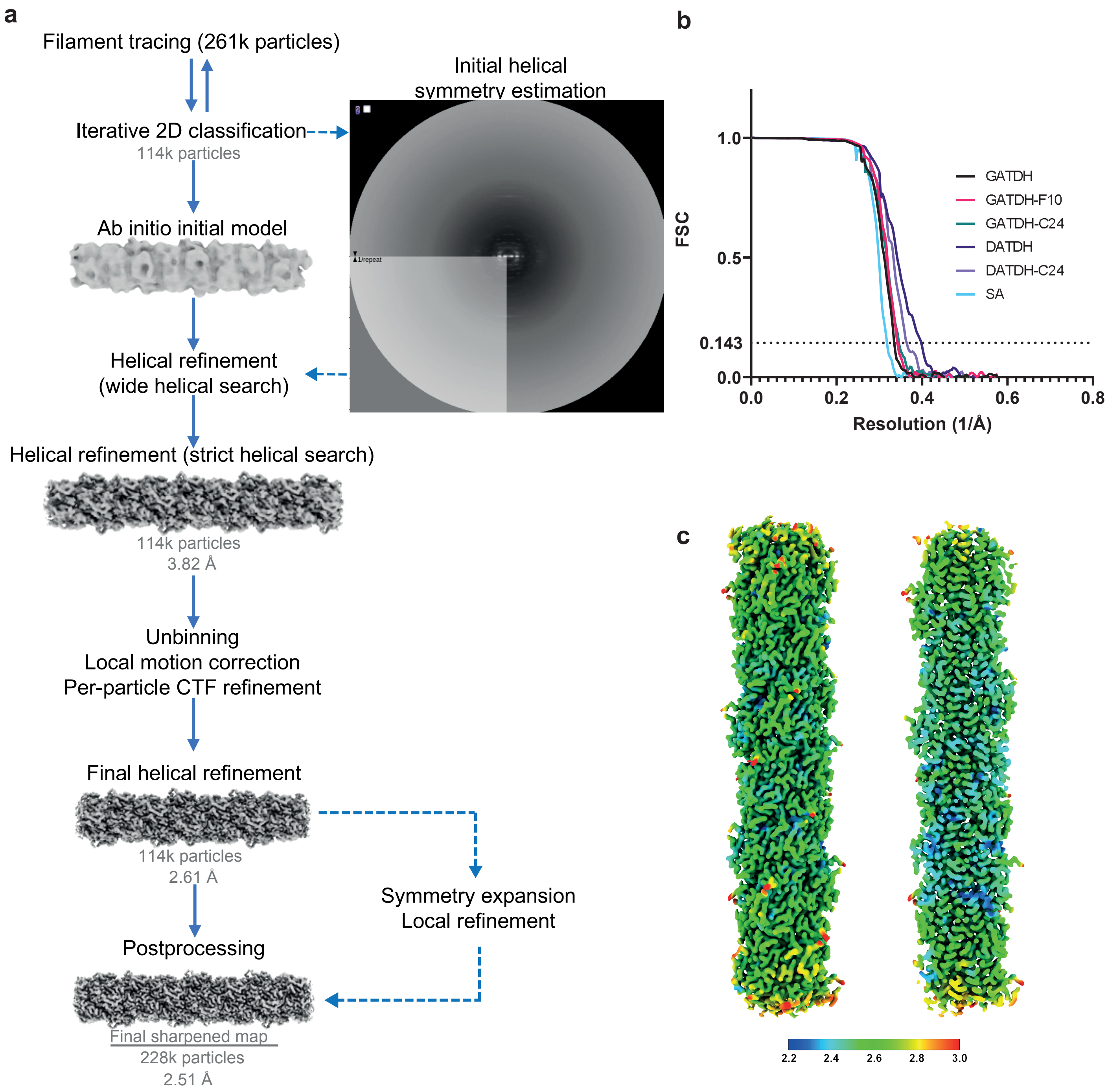

d

Sample	Twist (deg)	Rise (Å)
SA-GATDH	100,612	10,446
SB-GATDH	100,700	10,433
SB-GATDH-F10	100,700	10,449
SB-GATDH-C24	100,753	10,429
SB-DATDH-C24	100,706	9,956
SB-DATDH	100,683	9,977

Supplementary Fig. 2: CryoEM processing pipeline and parameters. a, Pipeline of cryoEM image processing. b, Fourier shell correlation (FSC) measurements on the 6 structures described in this manuscript. c, Representation of the resolution of structure on the surface of the pilus (left) and on its core, represented as a vertically clipped central section of the map (right). d, Twist and rise of the 6 different helical structures described here.

a

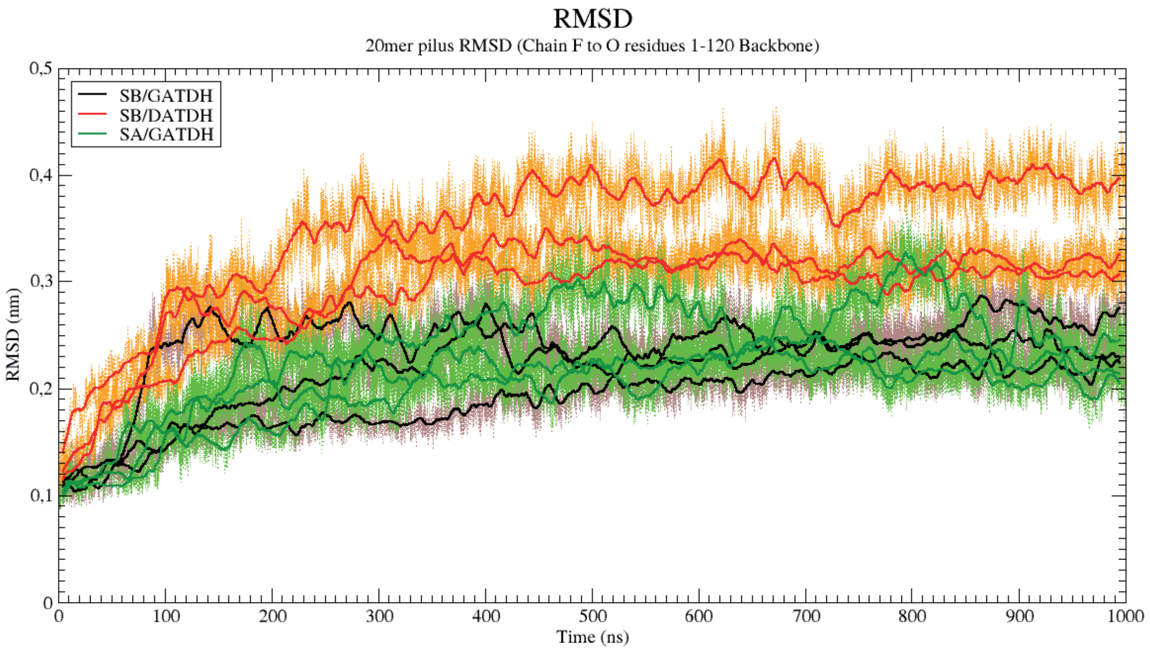

b

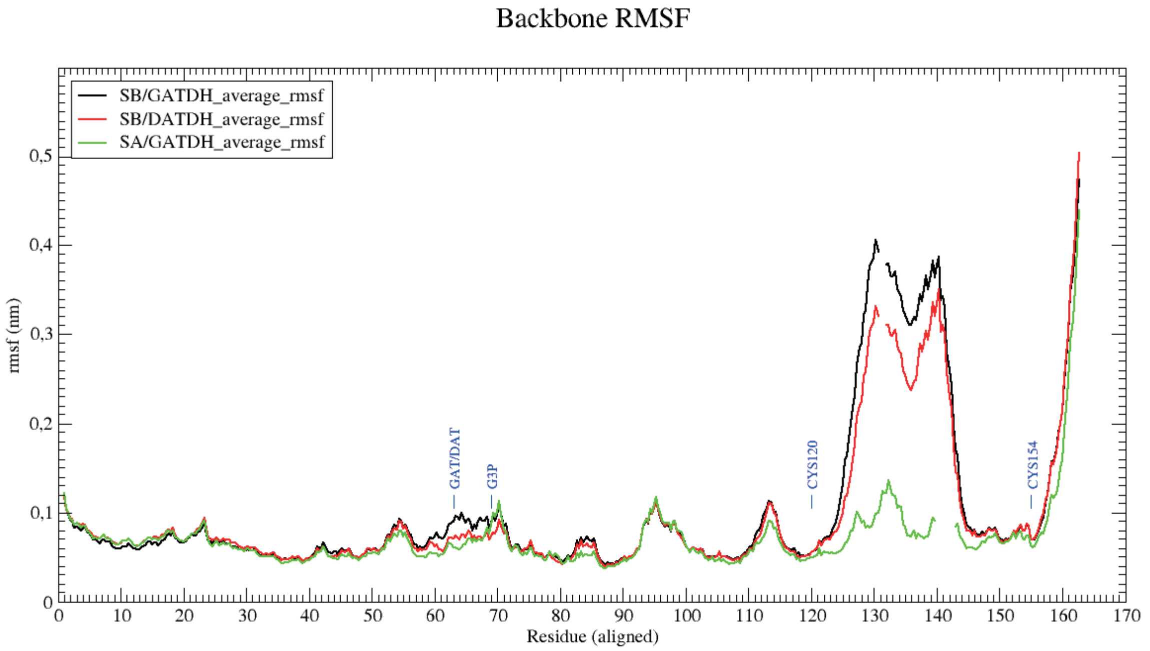

Supplementary Fig. 3: Molecular dynamics analysis of the pilus structures and their posttranslational modifications. a, Overall stability of the pilus structures during molecular dynamics analysis. Root-mean-square deviation of atomic positions (RMSD) of the following pilus structures are indicated, SB/GATDH (black), SB/DATDH (red) and SA/GATDH (green). RMSD was measured for residues 1-120, omitting the hypervariable loop. Three simulations of each are shown. b, Average backbone root-mean-square fluctuation (RMSF) of SB/GATDH (black), SB/DATDH (red), SA/GATDH (green), after sequence alignment (notice the gaps for SB or SA loops around positions ~131 or ~141, respectively). Positions of the GATDH/DATDH and G3P PTMs, and cysteines delimiting the hypervariable loop are marked in blue text.

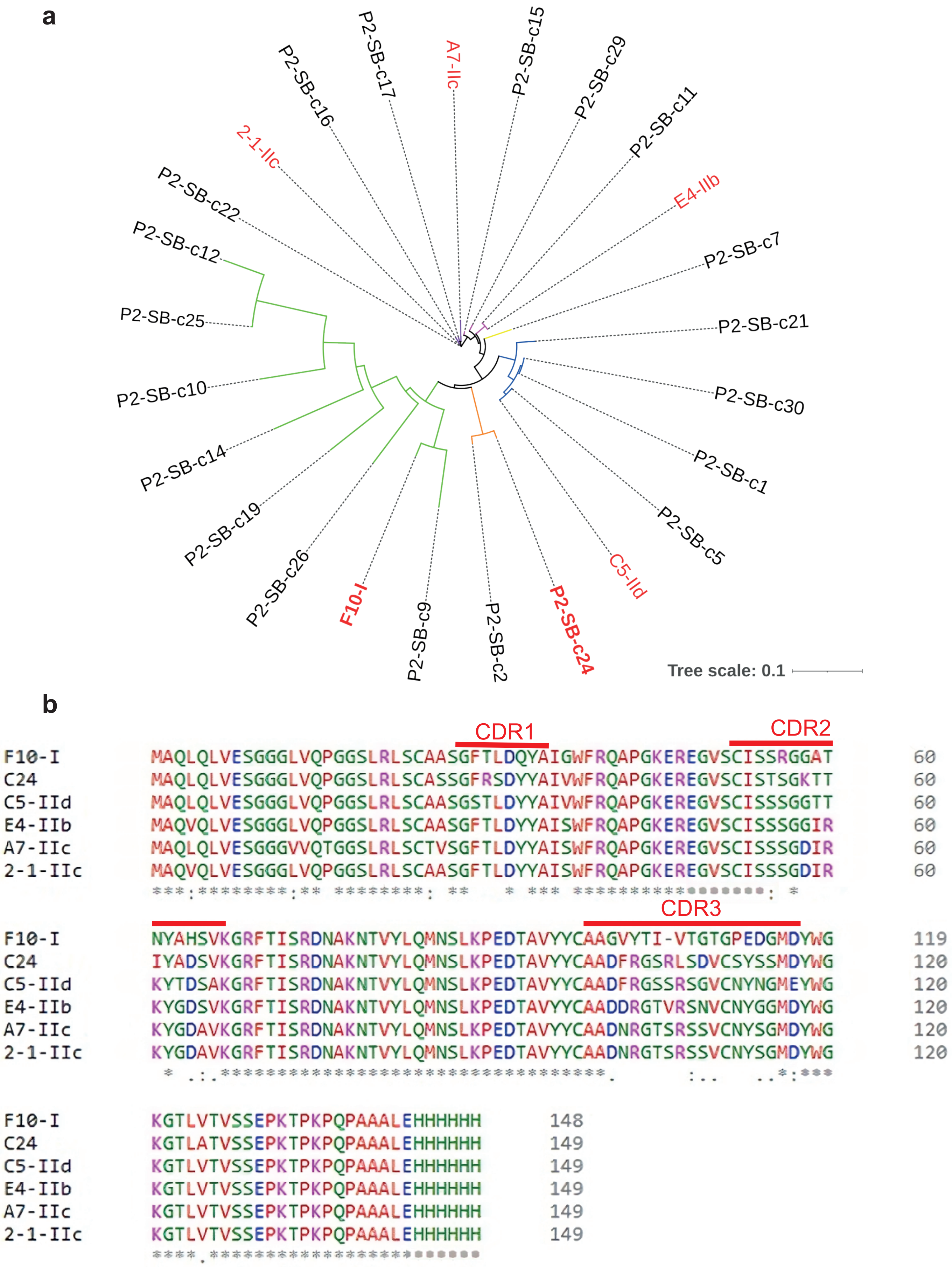

Supplementary Fig. 4: a, Phylogenetic tree of the 25 nanobody sequences resulting from the panning. Multiple alignment of the 25 nanobodies amino acid sequences was performed using Clustal Omega v1.2.4 59. Results were exported in Newick format and uploaded in the on-line tool iTol (interactive tree of life) to generate and edit the phylogenetic tree 60. Nanobodies selected for further studies are indicated in red. b, Sequence alignment of the 6 nanobodies selected for further studies.

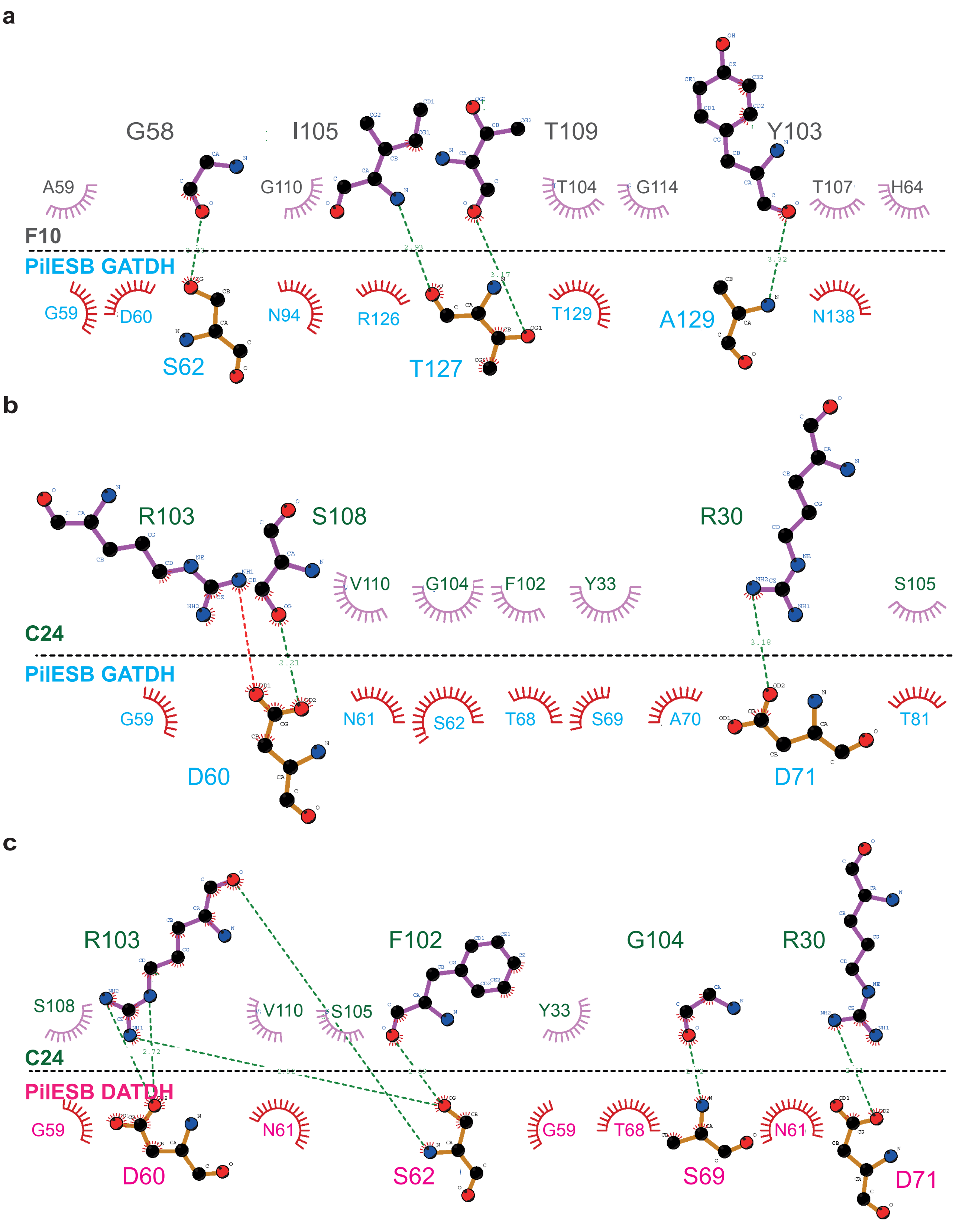

Supplementary Fig.5: Ligplots depicting interaction surface of nanobodies and pili. a, Interactions between PilESB-GATDH and the F10 nanobody. b, PilESB-GATDH and the C24 nanobody. c, PilESB-DATDH and the C24 nanobody.

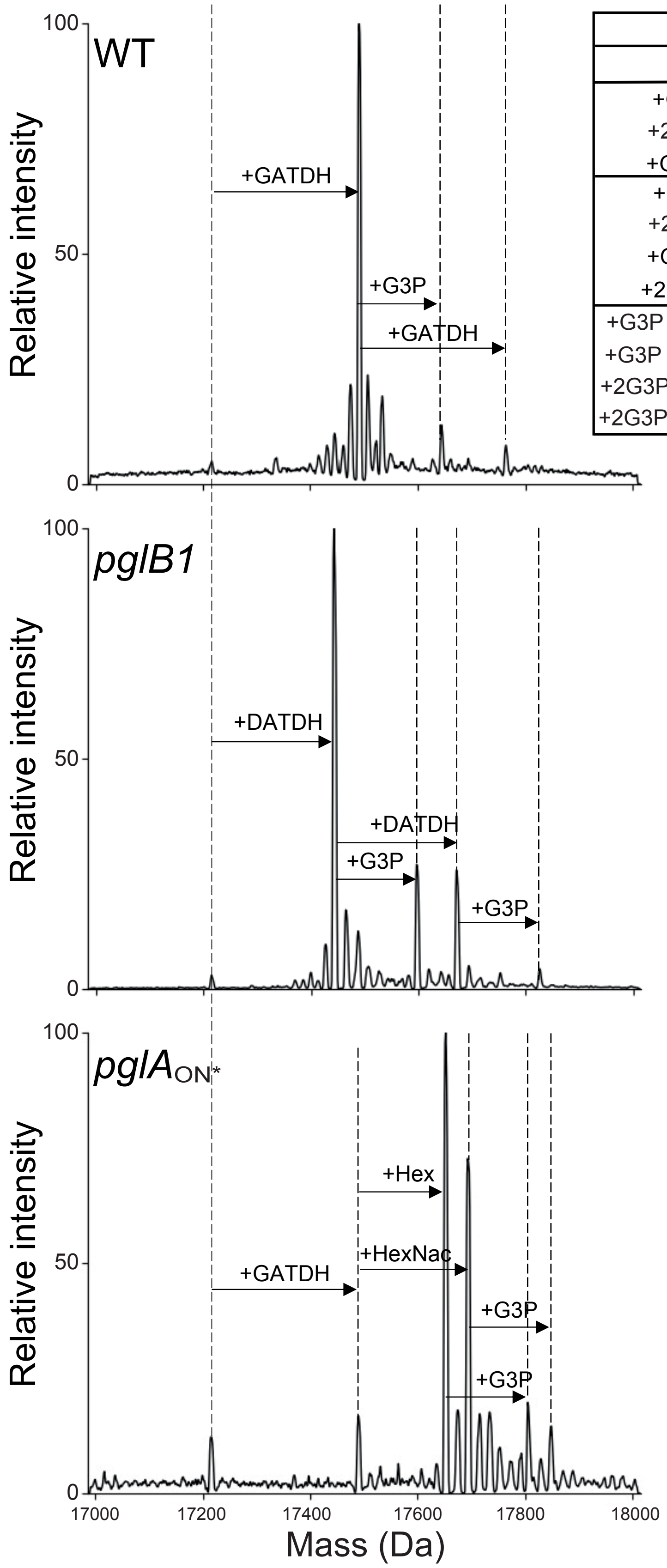

Composition	Mass (Da)	WT	*pglB1*	*pglA*$_{ON*}$
+G3P	17216.1	+	+	+
+G3P +GATDH	17490.4	+		+
+2G3P +GATDH	17644.1	+		
+G3P +2GATDH	17764.4	+		
+G3P +DATDH	17444.3		+	
+2G3P +DATDH	17598.1		+	
+G3P +2DATDH	17672.7		+	
+2G3P +2DATDH	17826.4		+	
+G3P +GATDH +Hexose	17652.1			+
+G3P +GATDH +HexNac	17694.3			+
+2G3P +GATDH +Hexose	17806.3			+
+2G3P +GATDH +HexNac	17848.1			+

GATDH: + 274.1 Da
DATDH: + 228.1 Da
G3P: + 154.1 Da

Supplementary Fig. 6: Mass spectrometry analysis of pilin monomers from the wild type (WT) strain, the *pglB1* mutant (*pglB1*) and the pglAON* strain (*pglAON**). Mass shifts due to pilin posttranslational modifications with sugars and phosphoglycerol are indicated with arrows on the graph. Intermediate peaks are due to salt adducts and possibly to minor forms of the pilin due to low levels of antigenic variation. A table provides the masses of each proteoform and their presence in the different strains.

! !

Supplementary Table 1. Cryo-EM data collection, image analysis, modelling, refinement, and validation statistics

**Cryo-EM data collection and image processing statistics**						
**Dataset**	SB-GATDH	SB-DATDH	SB-GATDH-F10	SB-GATDH-C24	SB-DATDH-C24	SA-GATDH
**Fitted model (PDB code)**	8P2V	8P36	8PJP	8PIJ	8PIZ	8P3B
**Cryo-EM Map (EMDB code)**	EMD-17375	EMD-17384	EMD-17718	EMD-17683	EMD-17695	EMD-17386
**Microscope**	Titan Krios G3i	Titan Krios G3i	Titan Krios G3i	Titan Krios G3i	Titan Krios G3i	Glacios
**Voltage (kV)**	300	300	300	300	300	200
**Detector**	K3	Falcon 4i	K3	Falcon 4i	Falcon 4i	Falcon 4
**Magnification**	105,000	130,000	105,000	130,000	130,000	130,000
**Pixel size (Å/pix)**	0.86	0.93	0.86	0.93	0.93	0.96
**Frames per exposure**	60	60	50	40	40	40
**Exposure (e^-/Å^2)**	50	60	61	40	40	40
**Defocus range (µm)**	-0.8 to -3.0	-0.6 to -2.6	-0.8 to -3.0	-0.6 to -3.0	-0.6 to -3.0	-1.0 to -3.0
**Micrographs collected/used**	5800/3315	1085/862	2622/1857	2994/2757	2502/1206	5034/3082
**Number of particles used/total**	271,827/469,659	114,167/261,469	158,939/686,009	434,690/770,309	249,060/270,780	1,127,987/2,001,170
**Map sharpening B-factor (Å^2)**	-106.4	-71.6	-108.7	-99.3	-82.8	-143.0
**Unmasked resolution at 0.5/0.143 FSC (Å)**	4.1/3.6	4.0/3.4	3.9/3.5	4.0/3.5	4.0/3.5	4.1/3.7
**Masked resolution at 0.5/0.143 FSC (Å)**	3.21/2.99	2.92/2.51	3.12/2.92	3.15/2.90	3.01/2.75	3.35/3.15

**Cryo-EM model refinement and validation**						
**Composition**						
**Amino acids**	161	161	286	288	288	159
**Atoms**	1226	1223	2158	2191	2188	1224
**RMSD bonds (Å) (# > 4σ)**	0.002 (0)	0.003 (0)	0.001 (0)	0.003 (0)	0.002 (0)	0.002 (0)
**RMSD angles (°) (# > 4σ)**	0.428 (0)	0.464 (0)	0.424 (0)	0.525 (0)	0.478 (0)	0.378 (0)
**Mean B-factors (Protein/Ligand)**	51.36/74.27	42.84/59.78	62.07/65.48	60.12/60.98	51.28/56.66	50.94/68.56
**Ramachandram**						
**Outliers (%)**	0.00	0.00	0.00	0.00	0.00	0.00

**Allowed (%)**	1.89	2.52	2.48	3.52	3.17	0.64
**Favoured (%)**	98.11	97.48	97.52	96.48	96.83	99.36
**Rotamer outliers**	3.94	5.51	0.89	3.90	5.63	0.00
**Cβ outliers (%)**	0.00	0.00	0.00	0.00	0.00	0.00
**CaBLAM outliers (%)**	0.64	1.91	1.08	2.14	1.43	1.29
**MolProbity score**	1.89	2.03	1.08	1.86	2.24	0.92
**Clash score**	2.47	6.60	2.11	3.93	8.79	1.65
**CC (mask)**	0.86	0.84	0.84	0.84	0.81	0.83

Supplementary Table 2

Strains used in this study

Strain	Genotype[a]	Reference
WT	8013	(Nassif *et al*, 1993)
WT GFP	8013 expressing GFP, Cm^{r}	(Soyer *et al*, 2013)
ΔG4	8013 ΔG4::Cm^{r}	(Kennouche *et al*, 2019)
pilD	8013 ΔG4::Cm^{r} *pilD ::*Kan^{r}	(Geoffroy *et al*, 2003)
pglD	8013 ΔG4::Cm^{r} *pglD::*Kan^{r}	(Geoffroy *et al*, 2003)
pptB	8013 ΔG4::Cm^{r} *pptB::*Sp^{r}	(Chamot-Rooke *et al*, 2011)
pglA$_{ON}$	8013 ΔG4::Cm^{r} *pglA*$_{ON}$*::*Kan^{r}	This study
pglB1	8013 ΔG4::Cm^{r} *pglB1::*Kan^{r}	This study
pilE$_{SA}$	8013 ΔG4::Cm^{r} *pilE*$_{SB}$*::pilE*$_{SA}$*::*Kan^{r}	(Kennouche *et al*, 2019)
pilTpilE$_{SA}$	8013 ΔG4::Cm^{r} *pilE*$_{SB}$*::pilE*$_{SA}$*::*Kan^{r} *pilT::*Ery^{r}	This study

[a] Cm, chloramphenicol; Km, kanamycin; Sp, spectinomycin; Ery, erythromycin; r, resistant

Primers used in this study

Primer name	Primer number	Sequence (5'→ 3')[a, b]
*pglB1*_MS11_fwd	SG7535776	ATTGGGTACCGGGCCCCCCCTCGAGGTTCGACATCATCGCATCC
*pglB1*_MS11_rev	SG7535777	TTTCGGATCCTTATGCCGTCCCGGTCTTG
Km_*pglB1*_fwd	SG7535778	GACGGCATAAGGATCCGAAAAGCAGCCG
Km-pglB1_rev	SG7535779	TATGTAATGGGGATCCAAAAGCAGCCGTC
HAD_*pglC* _fwd	SG7535780	TTTTGGATCCCCATTACATAATCAAACATGCAC
HAD_*pglC* _rev	SG7535781	AGGGAACAAAAGCTGGAGCTCTCGGCAAACACGGGATTTG
*pglA*_MC58_fwd	SG7562349	ATTGGGTACCGGGCCCCCCCTCGAGATTAAACCCCGACGACAAAG
*pglA*_MC58_rev	SG7562350	TTTCGGATCCAAATGACCGAAACTCAAAAGC
Km_*pglA*_fwd	SG7562351	TCGGTCATTTGGATCCGAAAAGCAGCCG
Km_*pglA*_rev	SG7562352	CCGTCCGCGCGGATCCAAAAGCAGCCGTC
*pglA*_2C43_fwd	SG7562353	TTTTGGATCCGCGCGGACGGATTCGGCATTC
*pglA*_2C43_rev	SG7562354	AGGGAACAAAAGCTGGAGCTCTGCTGTCCAACATCGCCGG
pFUSE_VHH_fwd	SG7648411	ACGAATTCGATATCGGCCATGGCTCAGTTGCAGCTCGTGG
pFUSE_VHH_rev	SG7648412	GTGGAGGAGATCTAGCGGCCGCTGGTTGTGGTTTTGGTGTC
*plgA*_mut-fwd	SG7732817	CGACAAATCACGCGG**c**GG**t**GG**c**GGCGATTTGGAACGG
C24mut_fwd	SG7783579	CTGAGCGACGTGTGC**gg**TTAT**gg**TTCCATGGACTACTG

[a] Restriction enzymes sites are underlined

[b] Mutations appear in bold

Plasmids used in this study

Plasmids	Properties	Reference
pET26b	Cloning and expression vector, kanamycin resistance	Novagen
pFuse-mIgG-Fc2	Cloning and expression vector, zeocin resistance	(Moutel *et al*, 2009)
pBluescript SK (+)	Cloning vector, ampicillin resistance	Stratagene
pET26b-F10-I	VHH F10 cloned into NcoI-NotI of pET26b, periplasmic expression	This study
pET26b-C24	VHH C24 cloned into NcoI-NotI of pET26b, periplasmic expression	This study
pET26b-$C24_{MUT}$	mutagenesis on pET26b-C24 ($S_{112}G$ $S_{114}G$)	This study
pFuse-muIgG-Fc2-F10	VHH F10 cloned into NcoI-NotI of pFuse-muIgG-Fc2	This study
pFuse-muIgG-Fc2-C24	VHH C24 cloned into NcoI-NotI of pFuse-muIgG-Fc2	This study
pFuse-muIgG-Fc2-$C24_{MUT}$	VHH $C24_{MUT}$ ($S_{112}G$ $S_{114}G$) cloned into NcoI-NotI of pFuse-muIgG-Fc2	This study
pBluescript SK (+)-*pglB1*	last 1219bp of *pglB1* from MS11-Kan^r-778bp downstream from *pglB2* of 8013	This study
pBluescript SK (+)-$pglA_{ON}$	last 667 bp of *pglA* from MC58 with 33bp intergenic region-Kan^r-700 bp downstream from *pglA* of 8013	This study
pBluescript SK (+)-$pglA_{ON*}$	mutagenesis on pBluescript SK (+)-$pglA_{ON}$	This study

Source data file for supplemtary figure 1c

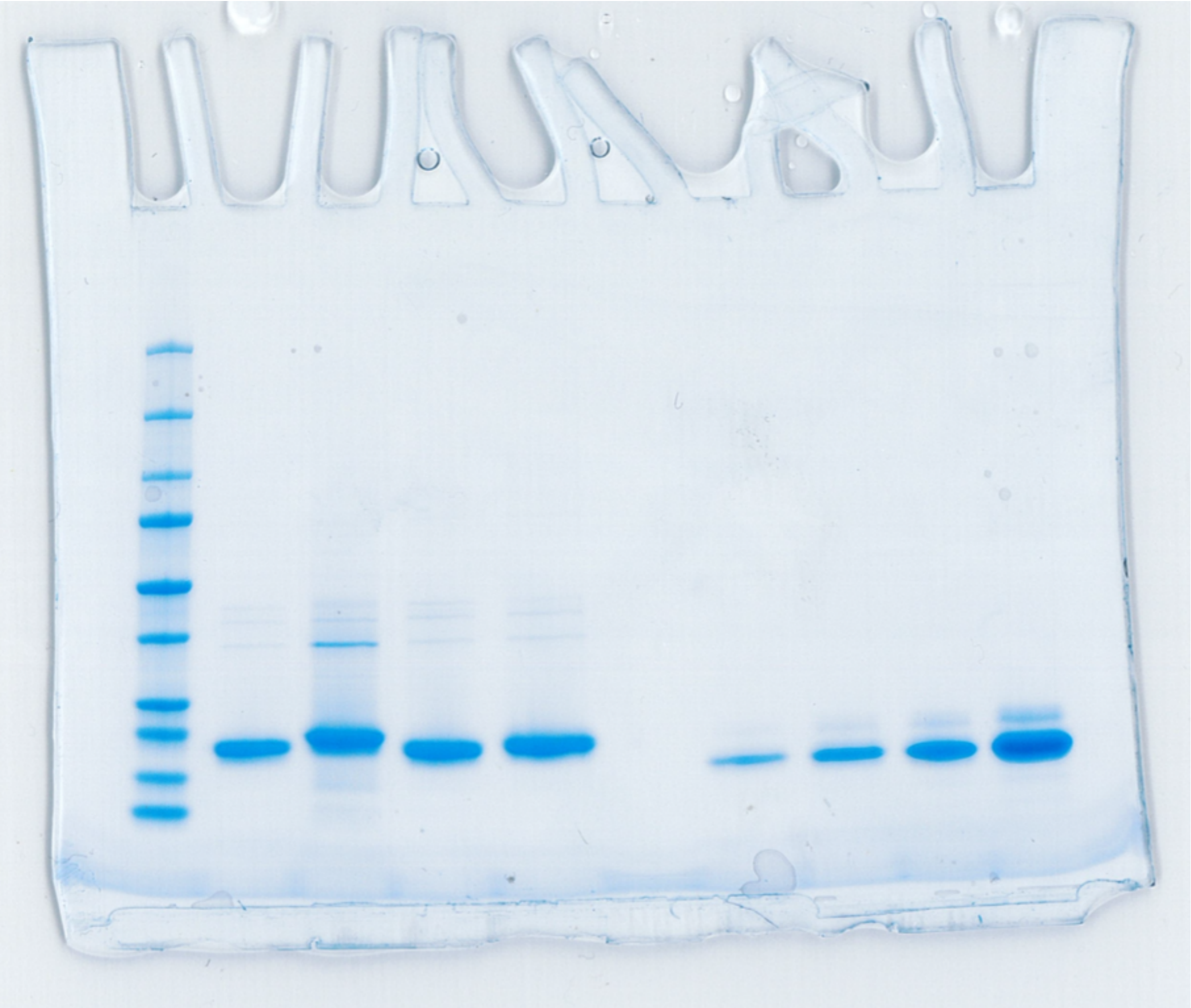

Supplement: Supplementary file 1 — Supplementary Information [file 41467_2024_46677_MOESM1_ESM.pdf]
