# Supplementary material for: Cryo-EM structures of type IV pili complexed with nanobodies reveal immune escape mechanisms
Source: Nat Commun. 2024 Mar 18;15:2414. doi: 10.1038/s41467-024-46677-y (PMC10948894; doi:10.1038/s41467-024-46677-y)
Supplement: Supplementary file 3 — Description of Additional Supplementary Files [file 41467_2024_46677_MOESM3_ESM.pdf]

Description of Additional Supplementary Files

File name: Supplementary Movie 1
Description: Molecular dynamics of a 20-mer pilus inserted in a lipid bilayer based on the structure described in Figure 1 over a 1µs period. The pilus is shown as cyan ribbon, and the POPE phospholipids are shown in a continuous fluid representation in transparent yellow (hydrophobic tails) and opaque red (polar heads).

File name: Supplementary Movie 2
Description: Molecular dynamics of a pilin monomer in the center of the pilus structure (10-mer). Front and side views are represented. The hypervariable loop is represented in dark blue. GATDH and G3P depicted as yellow and red sticks, respectively.

File name: Supplementary Movie 3
Description: Focus on the G3P part of the structure showing the formation of transient hydrogen bonds with surrounding amino acids. This video is a representative example chosen from the dynamics of 30 pilin monomers.

File name: Supplementary Movie 4
Description: Focus on the DATDH part of the structure showing the formation of stable hydrogen bonds with E56. This video is a representative example chosen from the dynamics of 30 pilin monomers.
