# Supplementary material for: Cryo-EM structures of type IV pili complexed with nanobodies reveal immune escape mechanisms
Source: Nat Commun. 2024 Mar 18;15:2414. doi: 10.1038/s41467-024-46677-y (PMC10948894; doi:10.1038/s41467-024-46677-y)
Supplement: Supplementary file 8 — Reporting Summary [file 41467_2024_46677_MOESM8_ESM.pdf]

nature portfolio

Corresponding author(s): Guillaume Duménil

Last updated by author(s): Feb 8, 2024

Reporting Summary

Nature Portfolio wishes to improve the reproducibility of the work that we publish. This form provides structure for consistency and transparency in reporting. For further information on Nature Portfolio policies, see our Editorial Policies and the Editorial Policy Checklist.

Statistics

For all statistical analyses, confirm that the following items are present in the figure legend, table legend, main text, or Methods section.

n/a	Confirmed	
☐	☒	The exact sample size (*n*) for each experimental group/condition, given as a discrete number and unit of measurement
☐	☒	A statement on whether measurements were taken from distinct samples or whether the same sample was measured repeatedly
☐	☒	The statistical test(s) used AND whether they are one- or two-sided<br>*Only common tests should be described solely by name; describe more complex techniques in the Methods section.*
☒	☐	A description of all covariates tested
☒	☐	A description of any assumptions or corrections, such as tests of normality and adjustment for multiple comparisons
☐	☒	A full description of the statistical parameters including central tendency (e.g. means) or other basic estimates (e.g. regression coefficient) AND variation (e.g. standard deviation) or associated estimates of uncertainty (e.g. confidence intervals)
☐	☒	For null hypothesis testing, the test statistic (e.g. *F*, *t*, *r*) with confidence intervals, effect sizes, degrees of freedom and *P* value noted<br>*Give P values as exact values whenever suitable.*
☒	☐	For Bayesian analysis, information on the choice of priors and Markov chain Monte Carlo settings
☒	☐	For hierarchical and complex designs, identification of the appropriate level for tests and full reporting of outcomes
☒	☐	Estimates of effect sizes (e.g. Cohen's *d*, Pearson's *r*), indicating how they were calculated

Our web collection on statistics for biologists contains articles on many of the points above.

Software and code

Policy information about availability of computer code

Data collection	EPU 2.0 and 3.0, Metamorph 7.10
Data analysis	Fiji, Motioncor2, PRISM 9, GCTF, HELIXPLORER-1, Alphafold2, Cryosparc 2.0-3.0, Relion 3.0-4.0, Coot 0.8-0.9, Chimera 1.06-1.14, ChimeraX 1.0-1.6, Phenix 1.14-1.21, Deepemhancer, CCP4 7.0-8.0, XMGRACE 5.1, GROMACS 2022.5, VMD, CHARMM, Unidec

For manuscripts utilizing custom algorithms or software that are central to the research but not yet described in published literature, software must be made available to editors and reviewers. We strongly encourage code deposition in a community repository (e.g. GitHub). See the Nature Portfolio guidelines for submitting code & software for further information.

Data

Policy information about availability of data

All manuscripts must include a data availability statement. This statement should provide the following information, where applicable:

- Accession codes, unique identifiers, or web links for publicly available datasets
- A description of any restrictions on data availability
- For clinical datasets or third party data, please ensure that the statement adheres to our policy

The cryo-electron microscopy data generated in this study have been deposited in the Electron Microscopy Databank under accession codes indicated below.

EMD-17375 [https://www.ebi.ac.uk/emdb/EMD-17375] (SB-GATDH structure)
EMD-17384 [https://www.ebi.ac.uk/emdb/EMD-17384] (SB-DATDH structure)
EMD-17718 [https://www.ebi.ac.uk/emdb/EMD-17718] (SB-GATDH-F10 structure)
EMD-17683 [https://www.ebi.ac.uk/emdb/EMD-17683] (SB-GATDH-C24 structure)
EMD-17695 [https://www.ebi.ac.uk/emdb/EMD-17695] (SB-DATDH-C24 structure)
EMD-17386 [https://www.ebi.ac.uk/emdb/EMD-17386] (SA-GATDH structure)

The structural data generated in this study have been deposited in the Protein Data Bank under accession codes indicated below.
8P2V [https://doi.org/10.2210/pdb8p2v/pdb] (SB-GATDH structure)
8P36 [https://doi.org/10.2210/pdb8p36/pdb] (SB-DATDH structure)
8PJP [https://doi.org/10.2210/pdb8pjp/pdb] (SB-GATDH-F10 structure)
8PIJ [https://doi.org/10.2210/pdb8pij/pdb] (SB-GATDH-C24 structure)
8PIZ [https://doi.org/10.2210/pdb8piz/pdb] (SB-DATDH-C24 structure)
8P3B [https://doi.org/10.2210/pdb8p3b/pdb] (SA-GATDH structure)

The input files, parameters and final snapshots for the Molecular Dynamics carried out in this study have been deposited in the Zenodo repository under accession codes indicated below.
10637491 [https://zenodo.org/uploads/10637491] (SB-GATDH, SB-DATDH, SA-GATDH MDs)

All mass spec data (raw and processed) are available via ProteomeXchange with accession code indicated below.
PXD046119 [https://www.ebi.ac.uk/pride/archive?keyword=PXD046119]

Research involving human participants, their data, or biological material

Policy information about studies with human participants or human data. See also policy information about sex, gender (identity/presentation), and sexual orientation and race, ethnicity and racism.

Reporting on sex and gender	NA
Reporting on race, ethnicity, or other socially relevant groupings	NA
Population characteristics	NA
Recruitment	NA
Ethics oversight	NA

Note that full information on the approval of the study protocol must also be provided in the manuscript.

Field-specific reporting

Please select the one below that is the best fit for your research. If you are not sure, read the appropriate sections before making your selection.

☒ Life sciences ☐ Behavioural & social sciences ☐ Ecological, evolutionary & environmental sciences

For a reference copy of the document with all sections, see nature.com/documents/nr-reporting-summary-flat.pdf

Life sciences study design

All studies must disclose on these points even when the disclosure is negative.

Sample size	Sample size for mouse experiments (Fig. 3d) were based on previous studies (eg Melican et al PLOS Path 2013), and all efforts were made to minimize the number of animals involved.
Data exclusions	No data was excluded
Replication	All experiments were repeated 3 times independantly, for ELISA and aggregation assays 3 replicates were performed in each experiment.
Randomization	Animals were randomly distributed in different groups. For ELISA and aggregation assays samples were also allocated randomly.
Blinding	All measurements were quantitative and not submitted to person to person varation. Elisa assays are read by a microplate reader, aggregation assays by automated image analysis, results of animal studies depend on CFU counts

Reporting for specific materials, systems and methods

We require information from authors about some types of materials, experimental systems and methods used in many studies. Here, indicate whether each material, system or method listed is relevant to your study. If you are not sure if a list item applies to your research, read the appropriate section before selecting a response.

Materials & experimental systems

n/a	Involved in the study
☐	☒ Antibodies
☐	☒ Eukaryotic cell lines
☒	☐ Palaeontology and archaeology
☐	☒ Animals and other organisms
☒	☐ Clinical data
☒	☐ Dual use research of concern
☒	☐ Plants

Methods

n/a	Involved in the study
☒	☐ ChIP-seq
☒	☐ Flow cytometry
☒	☐ MRI-based neuroimaging

Antibodies

Antibodies used	The following antibodies were used for immunofluorescence: mouse monoclonal antibody anti-PilE, clone 20D9 (1µg/ml, Pujol et al, 1999) and mouse anti-His tag (3.5 µg/ml, Biolegend, #BLE906102, clone J095G46). The following goat secondary antibodies were used for immunofluorescence and enzyme-linked immunosorbent assay (ELISA): anti-mouse IgG (H+L) coupled to Alexa Fluor 488 or 568 (1/200, Invitrogen, #A21121 and #A11004), F(ab')$_2$ Fragment Goat Anti-Mouse IgG Fcγ fragment coupled to horseradish peroxidase (1/2500, Jackson ImmunoResearch, #115-036-071).
Validation	The 20D9 anti pilin antibody which was validated in Pujol et al 1999. We have also verified its specificity by immunofluorescence on WT and pilE mutant strains. Please refer to manufacturers for the validation of other antibodies.<br>The mouse anti-his tag monoclonal antibody was tested against his-tagged proteins by western blot and by immunofluorescence on HeLa cells transfected with his-tagged proteins.

Eukaryotic cell lines

Policy information about cell lines and Sex and Gender in Research

Cell line source(s)	Primary Human umbilical vein endothelial cells (HUVEC) were purchased from Promocell
Authentication	These are primary cells directly isolated from umbilical cords by Promocell. There is therefore no need for authentification
Mycoplasma contamination	Cells were checked regularly by immunofluorescence following DNA staining (DAPI). The presence of Mycoplasma is evident when staining is seen outside of the cell nucleus.
Commonly misidentified lines (See ICLAC register)	No commonly misidentified cell line has been used in this study

Animals and other research organisms

Policy information about studies involving animals; ARRIVE guidelines recommended for reporting animal research, and Sex and Gender in Research

Laboratory animals	SCID/Beige (CB17.Cg-PrkdcscidLystbg-J/Crl), males and females, aged between 18 and 22 weeks were used. One young adult male alpaca (Lama pacos) was immunized.
Wild animals	No wild animals were used in this study.
Reporting on sex	Male or female mice were used randomly. The single Alpaca used here was a male.
Field-collected samples	No field collected samples were used in this study.
Ethics oversight	Experiments involving all animals were evaluated by the local ethical comity at Institut Pasteur, the CETEA.

Note that full information on the approval of the study protocol must also be provided in the manuscript.

Plants

Seed stocks	NA
Novel plant genotypes	NA
Authentication	NA
